# Preeclampsia: Cardiotonic Steroids, Fibrosis, Fli1 and Hint to Carcinogenesis

**DOI:** 10.3390/ijms22041941

**Published:** 2021-02-16

**Authors:** Natalia I. Agalakova, Nikolai I. Kolodkin, C. David Adair, Alexander P. Trashkov, Alexei Y. Bagrov

**Affiliations:** 1 Sechenov Institute of Evolutionary Physiology and Biochemistry, 44 Torez Prospect, 194223 St. Petersburg, Russia; nagalak@mail.ru; 2 State Institute of Highly Pure Biopreparations and Sechenov Institute of Evolutionary Physiology and Biochemistry, 44 Torez Prospect, 194223 St. Petersburg, Russia; n.i.kolodkin@hpb.spb.ru or; 3 Department of Obstetrics and Gynecology, University of Tennessee, Chattanooga, TN 37402, USA; David@solasbio.com or; 4 Konstantinov St. Petersburg Nuclear Physics Institute, National Research Centre Kurchatov Institute, 1 Orlova Roshcha, 188300 Gatchina, Russia; alexandr.trashkov@gmail.com

**Keywords:** preeclampsia, Na/K-ATPase, marinobufagenin, Fli1, TFG-beta, collagen-1, vascular fibrosis

## Abstract

Despite prophylaxis and attempts to select a therapy, the frequency of preeclampsia does not decrease and it still takes the leading position in the structure of maternal mortality and morbidity worldwide. In this review, we present a new theory of the etiology and pathogenesis of preeclampsia that is based on the interaction of Na/K-ATPase and its endogenous ligands including marinobufagenin. The signaling pathway of marinobufagenin involves an inhibition of transcriptional factor Fli1, a negative regulator of collagen synthesis, followed by the deposition of collagen in the vascular tissues and altered vascular functions. Moreover, in vitro and in vivo neutralization of marinobufagenin is associated with the restoration of Fli1. The inverse relationship between marinobufagenin and Fli1 opens new possibilities in the treatment of cancer; as Fli1 is a proto-oncogene, a hypothesis on the suppression of Fli1 by cardiotonic steroids as a potential anti-tumor therapeutic strategy is discussed as well. We propose a novel therapy of preeclampsia that is based on immunoneutralization of the marinobufagenin by monoclonal antibodies, which is capable of impairing marinobufagenin-Na/K-ATPase interactions.

## 1. Introduction

Preeclampsia (PE) is one of the most common hypertensive disorders developing during pregnancy [1]. Affecting approximately 2–8% of pregnancies worldwide, it increases the risk of complications for both mothers and babies [2]. Despite the ongoing prophylaxis and repeated attempts to select a therapy, it is still not possible to reduce the incidence of PE. As a result, it is one of the leading causes of maternal and perinatal morbidity and mortality. The major clinical manifestation of PE is increased blood pressure (≥140/90 mmHg registered in at least two measurements with a 6 h interval) after 20 weeks gestation [1]; however, the definite diagnosis of PE requires a combination of such features as hypertension, proteinuria (the presence of ≥300 mg of protein/L of urine in a daily sample or in two samples taken six hours apart), generalized edema and any associated organ dysfunction [2,3].

Despite many investigations, the etiology of PE is not completely understood. The pathophysiological mechanisms of PE revealed to date include placental abnormalities or injury, general endothelial dysfunction, abnormal production of angiogenic factors and vasoactive substances, placental steroid and peptide hormones, oxidative stress, insulin resistance and a disturbed immune interaction between the maternal organism and placenta [4,5]. The final result of this complex interaction is a multisystem disorder characterized by hypertension, proteinuria and multiple end-organ ischemia or dysfunction. The present review is focused on the mechanisms related to the abnormal processes in blood vessels and presents the “fibrotic” concept of PE development. This model postulates that one of the major causes of PE is linked with an enhanced synthesis of cardiotonic steroids (CS), which leads to disturbances in intracellular signaling and finally to altered vascular reactivity including arterial fibrosis. The immunoneutralization of CS as a valuable approach to relieve the symptoms of PE and a promising therapy are discussed as well.

## 2. Bufadienolide Cardiotonic Steroids

The search for candidates for the role of natriuretic hormones has found several circulating steroid compounds represented by cardenolides that include ouabain and digoxin and bufadienolides such as marinobufagenin [6]. Bufadienolide CS differ from cardenolides by the presence of a twice-unsaturated six-membered lactone ring. For several decades it has been known that some representatives of amphibians, for example, the toad Bufo marinus, can synthesize the steroids of bufadienolide group [7]. The endogenous level of bufadienolides in toads increases following their migration from arid habitats to areas with high humidity [7]. As amphibian skin is the most important organ involved in the regulation of the water-salt balance, it has been suggested that the sodium pump and bufadienolides are physiological regulators of sodium transport [8]. A hormone with mass spectral characteristics identical to the one of amphibian bufadienolides, marinobufagenin (MBG), was isolated from the urine of patients with myocardial infarction [9]. In the in vitro and in vivo experiments, monoclonal antibodies to MBG restored the activity of Na/K-ATPase [10]. It was shown later that MBG is synthesized by the cells of the adrenal cortex and placenta by the transformation of bile acids using one of the enzymes of the P450 cytochrome family, CYP27A1 [11]. MBG selectively interacts with the alpha-1 isoform of Na/K-ATPase, the main isoform of the enzyme in the kidneys and blood vessels, and it is an active vasoconstrictor and natriuretic [6,12]. The content of MBG in blood plasma increases with the rise in volume of circulating fluid and with sodium retention; for example, in patients with hypertension and chronic renal failure as well as with congestive circulatory failure [13,14].

## 3. Cardiotonic Steroids, Pregnancy and Preeclampsia

As a normal pregnancy is accompanied by fluid retention and a positive sodium balance in the body [15], interest in the possible role of CS in pregnancy arose shortly after their discovery. Graves et al. showed that the level of digoxin-like immunoreactivity increases moderately in a healthy pregnancy and rises significantly with gestational hypertension [16]. Based on these observations, the authors suggested that excessive CS synthesis may be one of the factors implicated in PE pathogenesis [16]. Over the next few years, the findings of Graves et al. found their confirmation in the works of other clinicians [17,18,19,20]. Convincing evidence of the contribution of CS to the pathogenesis of PE was obtained in experimental and clinical studies using the Digibind preparation, which is a lyophilized Fab fragment of affinity-purified sheep anti-digoxin antibodies and has cross-immunoreactivity with several CS [21,22]. The administration of Digibind caused a decrease in blood pressure in rats with volume-dependent hypertension, accompanied by an increase in the concentration of CS in blood plasma [23]. During pregnancy, the site of synthesis of CS is the placenta [24]. Di Grande et al. showed that Digibind at a concentration of 130 μg/ml causes a significant decline in the vascular tone of perfused placentas obtained after delivery from patients with PE [25]. Armler et al. found that the development of PE is accompanied by a significant increase in the sensitivity of placental Na/K-ATPase to digitalis preparations [26]. Moreover, it was the cells of placental origin, JEG-3, which were found to synthesize MBG from the bile acids using the enzyme CYP27A1 [11].

Clinical studies suggest that one of the mechanisms of PE pathogenesis is the excessive production of MBG. Thus, the MBG content in the plasma of pregnant patients with severe PE (blood pressure 161/104 mmHg) was enhanced by five times compared with that in women with an uncomplicated pregnancy while in the case of moderate PE (blood pressure 149/93 mmHg), by two times [27,28]. It was later shown that such a two-fold increase in the level of MBG in plasma following the development of moderate PE was accompanied by a 50% inhibition of the activity of Na/K-ATPase in red blood cells whereas antibodies to MBG, unlike antibodies to ouabain, restored the activity of enzymes ex vivo [28]. These observations indicate that MBG is a factor responsible for the suppression of Na/K-ATPase activity in PE as well as a marker of the severity of this syndrome. In in vitro experiments, MBG in the “pathophysiologically significant” concentration range (1–3 nmol/L) triggered a contractile response in the isolated rings of the human mesenteric arteries and induced a 25% inhibition of Na/K-ATPase in arterial tissue [27,28]. Therefore, MBG levels observed in patients with PE in vivo can increase the vascular tone and significantly inhibit Na/K-ATPase [28].

## 4. Fibrosis and Preeclampsia

**Figure 1:**
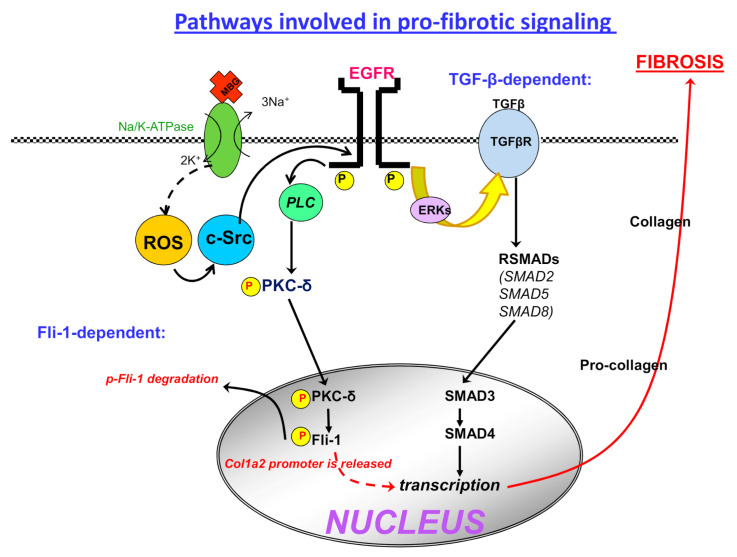
Schema of “signaling” pathways for CS effects. The “signaling” pathway involves the association of Src with the Na/K-ATPase. Binding of the CS to the Na/K-ATPase activates Src, which transactivates the epidermal growth factor receptor (EGFR) and phospholipase C (PLC). This leads to a generation of cascades that involve the generation of PKC-δ and activation of Fli1 or the activation of TGF-β and SMAD and finally the activation of collagen-1 and fibrosis.

During PE, the spiral arteries of the placenta lose their elastic properties, which ultimately leads to poor placental perfusion [29,30]. This phenomenon was established to be associated with another important effect of CS, which was their ability to function as pro-fibrotic factors. Recent studies have shown that in addition to the vasoconstrictor effect, CS are important regulators of intracellular signaling cascades leading to a loss of elasticity and vascular fibrosis [31,32]. Both effects of CS interacting with each other are associated with the remodeling of myocardial tissue and blood vessels along with other processes leading to the deposition of collagen and the impaired ability of vessels to relax [33]. Recently, it was shown that nanomolar concentrations of MBG stimulate collagen synthesis and induce fibrosis in the cardiovascular and kidney tissues [33,34]. A culture of the fibroblast with CS was shown to enhance the collagen synthesis in cells, which confirmed the signaling function of the MBG-Na/K-ATPase complex, which was different from the classical function of the Na/K-ATPase (Figure 1) [32]. In vivo administration of MBG to rats at a concentration observed in the plasma of patients with renal failure caused myocardial fibrosis accompanied by the increased expression of Src kinase and phosphorylation of one of the mitogen-activated protein kinases (MAPK) ERK1/2 in the myocardium [32]. One of the most important mechanisms underlying the pro-fibrotic effect of MBG is the altered activity of Fli1, a nuclear transcription factor and a negative regulator of collagen-1 synthesis [33,34]. The inhibition of Fli1, a member of the erythroblast transformation specific (ETS) family, is critical for MBG-induced fibrosis [32]. Fli1 acts as a negative regulator of collagen-1 synthesis and it competes with another transcription factor, ETS-1, to maintain a balance between stimulation and repression of the collagen-1 gene [35,36]. The Na/K-ATPase/Src/EGFR complex begins a signal cascade, which activates phospholipase C (PLC) resulting in the phosphorylation of PKCδ and its translocation to the nucleus. In the nucleus, phosphorylated PKCδ phosphorylates Fli1, which withdraws the Fli1-induced inhibition of the collagen-1 promoter and increases procollagen expression and collagen production [32] (Figure 1). The mechanism of vascular fibrosis induced by various vascular factors including CS is also implicated in the remodeling of spiral uterine arteries associated with the development of PE [32,37]. Interestingly, the same mechanism of synthesis of collagen-1, Fli1-dependent fibrosis, was found in the myocardium of rats with renal failure [38].

In our study, performed on umbilical arteries obtained after delivery from patients suffering from PE, the collagen content in vessels was much higher but the level of Fli1 was lower compared with those in the arteries of women with an uncomplicated pregnancy and the arteries themselves were less sensitive to the relaxing effect of sodium nitroprusside [33,34]. The suppression of Fli1 and excessive synthesis of collagen-1 in the placenta and umbilical arteries obtained from the patients with PE was confirmed by another recent study [37]. Previously, it was also shown that the blood plasma of women with PE contains an increased amount of MBG [27,28]. In pregnant rats, an increase in the MBG content caused by the consumption of NaCl was accompanied by the development of typical symptoms of PE including increased blood pressure, proteinuria and a decreased weight and size of fetuses [39]. Taking into account that MBG stimulates the synthesis of collagen, the development of fibrosis in the placenta and umbilical arteries of patients with PE is accompanied by the increased production of MBG and a substantial suppression of Fli1 [36], as well as that, in PE, vascular stiffness is based on elevated MBG levels [33,40], one can conclude that MBG is one of the major factors involved in the pathogenesis of PE through the induction of vascular fibrosis. In addition, it is assumed that blood vessels exposed to the negative effects of PE factors are subsequently more sensitive to damage despite the disappearance of symptoms of PE after the delivery [41]. It should be noted that the Fli1-dependent mechanism is not a single mechanism of MBG-induced fibrosis. In several experimental models including salt-loaded young Sprague Dawley rats and salt-sensitive Dahl rats MBG exerted its pro-fibrotic effect acting via the activation of TGF-β, the SMAD2-3 signaling pathway and activation collagens-1,-2,-3,-4 and -5 [42,43]. Interestingly, in experimental salt-loaded rats with type-2 diabetes mellitus, both mechanisms of fibrosis in the aorta and myocardium, Fli1- and TGF-β-dependent, were taking place [44].

## 5. Immunoneutralization of Cardiotonic Steroids

Besides its effects on vascular tone, the action of MBG has been associated with vascular fibrosis [34]. Fli1 acts as a negative control of collagen-1 synthesis and it competes with another transcription factor, ETS-1, to maintain a balance between the stimulation and repression of the Col1a1 gene [35]. The inhibition of Fli1, a nuclear transcription factor and a member of the ETS family, is implicated in MBG-induced fibrosis [32]. MBG activates a Na/K-ATPase/Src/EGRF complex (Figure 1) and initiates a signal cascade that activates PLC resulting in the phosphorylation of PKC-δ and its translocation to the nucleus. In the nucleus, phosphorylated PKC-δphosphorylates Fli1, which leads to a more rapid catabolism of Fli1, a negative regulator of collagen-1 synthesis. The removal of the Fli1-induced inhibition of the Col1 gene promoter increases the procollagen expression and collagen production [32]. The similarity of the structure of cardenolides (digoxin, ouabain) and bufadienolides (MBG) has allowed the use of polyclonal antibodies against digoxin (Digibind and Digifab) for the treatment of PE in clinical practice [45,46]. Digibind has been used for many years to relieve the severity of symptoms in patients after poisoning with digoxin [47]. Over the past 20 years, digoxin-specific Fab fragments (Digibind) have been successfully used to treat the intoxication with bufadienolides of toads and be effective in animals and humans [48,49]. The administration of Digibind was shown to decrease blood pressure in animals with experimental hypertension due to the interaction with an endogenous digoxin-like factor [50]. In 1988, Goodlin achieved a persistent decrease in blood pressure in a pregnant woman with PE that developed at 26 weeks of gestation after a twice-intravenous administration of Digibind at a dose of 0.087 mg/kg [19]. Graves and co-authors, using a Digibind-based immunoassay, demonstrated the placental origin of human CS and showed that ketoconazole, a steroid synthesis inhibitor, inhibited its biosynthesis [51]. Importantly, placental hypoxia was shown to increase placental CS release [51]. Adair et al. observed a decrease in blood pressure in a patient with PE on the background of a combined intravenous bolus of 5 mg Digibind and a 24 h infusion of the drug (1 mg per hour) [52]. Somewhat later, the effectiveness of a single administration of Digibind was confirmed in a double-blind, placebo-controlled study in 13 patients with PE developed postpartum [53]. Another positive finding was that in patients with PE, treatment with Digibind resulted in an increase of creatinine clearance vs. that in the placebo group [54]. A multicenter study of the effectiveness of Digibind for the treatment of severe PE completed in 2007 showed that the administration of Digibind led to a significant increase in creatinine clearance and decreased the risk of pulmonary edema compared with the patients who did not receive antibodies [55]. It is noteworthy that in the above studies Digibind did not cause any side effects in patients. However, in 2011, the production of Digibind was discontinued and Digifab (BTG International Ltd., United Kingdom) was the only digoxin antibody preparation registered with the US Food and Drug Administration. A comparative study of Digifab and Digibind showed that they have comparable cross-reactivity with bufadienolides and cardenolides [56]. In patients with PE, an increase in the immunoreactivity to MBG was identical for both Digifab and Digibind and both antibodies restored Na/K-ATPase activity [56].

**Figure 2:**
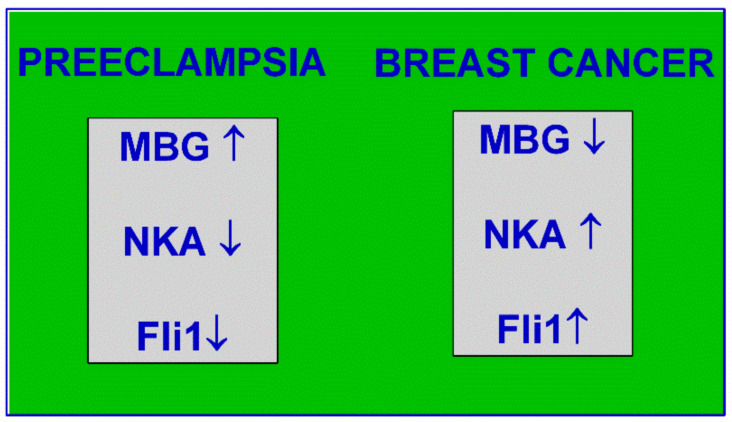
Preeclampsia (left) and breast cancer (right) are associated with different marinobufagenin (MBG)–Na/K-ATPase–Fli1 scenarios.

MBG stimulates collagen synthesis in vascular smooth muscle cells and MBG immunoneutralization with specific antibodies in preeclampsia leads to a decrease in collagen-1 levels. The stimulation of the synthesis of collagen-1 by MBG is based on the inhibition of the nuclear transcription factor Fli1, which is a suppressor of the promoter of the Col1 gene responsible for the synthesis of procollagen-1 due to the activation of protein kinase C. Thus, the silencing of the Fli1 gene using inhibitory RNA should lead to consequences comparable with those in not only preeclampsia and chronic kidney diseases but also in advanced hypertension. A recent work also showed that the incubation of explants of umbilical arteries obtained from the patients with PE with monoclonal antibodies against MBG led to a significant decrease in the collagen-1 content [37]. The incubation of healthy human umbilical arteries in the presence of low MBG concentrations for 24 h led to a decline in the Fli1 content and an increase in the PKC-delta expression while the level of procollagen-1 synthesis increased six-fold [37]. Another work has shown that the introduction of humanized anti-MBG monoclonal antibodies leads to phosphorylation of MAP kinase p38 in cytotrophoblast cells, indicating a possible therapeutic effect of these antibodies [57]. Moreover, a recent study has demonstrated that the silencing of Fli1 in human umbilical arteries mimics preeclamptic phenotypes through activating PKCδ and the activation of procollagen and collagen-1 synthesis [57,58]. The antagonism of endogenous CS can be one of the new and fundamentally different possibilities of pharmacological therapy and prevention of vascular fibrosis. Therefore, a question remains. Is the absence of CS-induced Na/K-ATPase inhibition such an innocent entity? In PE, when CS are elevated, Na/K-ATPase is depressed and Fli1 is low this results in the occurrence of a phenotype associated with a significantly lower incidence of breast cancer [59,60] (Figure 2). At the same time in breast cancer patients (Figure 2) CS and Na/K-ATPase are regular [61,62,63,64,65] and Fli1 is high [66,67,68]; i.e., as if patients with PE administered an anti-CS antibody. Could it be true for the other types of cancer?

## 6. Interaction of CS and Fli1 and a Hint for Cancer

As mentioned previously, when antibodies to CS are administered to experimental animals or are applied to tissues from pregnant humans, the concentrations of Fli1 in the exhibit increases [33,34]. The levels of MBG in plasma are in a reciprocal relationship with the level of Fli1, a member of the ETS family, and the anti-fibrotic factor [32]. When levels of MBG are increased, levels of Fli1 decrease and a Col-1 gene promoter is released from the nucleus and procollagen-1 and collagen become activated [32]. While many studies have demonstrated that Fli1 is a pro-cancer factor [68,69], CS including MBG are becoming attractive anti-cancer drug candidates [70,71]. It is generally accepted that PE is associated with a low risk of cancer, which is not surprising considering that endogenous levels of MBG and other CS are dramatically increased in PE patients [27,28] and therefore suppress the growth of tumors in vivo and in vitro [72,73,74]. Therefore, theoretically, in cancer patients and experimental animals, MBG should suppress the levels of Fli1, an oncogene. This notion is agreement with the data showing the levels of Na/K-ATPase inhibitors measured in breast cancer patients. Weidemann found that a majority (73.6%) of 84 patients expressed lower CS plasma concentrations (< 50 pmol/L) than that in the control group (150 pmol/L) [75,76]. This observation was confirmed quite recently and when levels of endogenous bufalin in the serum of patients with hepatocellular carcinoma were compared with controls they were four times lower vs. control levels [77,78,79].

Fli1 belongs to the ETS family, one of the large family of transcription factors that are highly conserved and are unique to animals [80,81]. The ETS family is involved in a wide variety of functions including the regulation of cellular differentiation, control, cell proliferation, apoptosis and angiogenesis and is associated with cancer [82]. Fli1 is encoded by the *FLI1* gene, which is a proto-oncogene. Fli1 was first identified in cancer, systemic sclerosis and tissue fibrosis [83,84]. This phenotype was consistent with the role of Fli1 as a regulator of vessel maturation; thus, in rats following a subtotal nephrectomy, elevated MBG led to a reduction in the level of Fli1 and an increase in the collagen-1 level in the myocardium. A single administration of a monoclonal anti-MBG antibody in rats produced an anti-fibrotic effect; that is, restored Fli1 levels and a reduced collagen-1 abundance in the myocardium were observed [38]. Fli1 attracted attention primarily because of its contribution to different types of cancer including gastric cancer, Burkitt lymphoma, breast cancer, pancreatic ductal adenocarcinoma, small cell lung cancer and Ewing’s sarcoma [57,85,86,87]. We observed extremely high levels of MBG and low levels of Fli1 along with an extremely high level of collagen-1 in patients and experimental animals with preeclampsia, chronic renal failure and malignant hypertension [33,37,38]. When animals from all three groups were given a 3E9 monoclonal antibody against MBG it was associated with an increase in Fli1 and a dramatic reduction of fibrosis, suggesting that CS are potentially anti-cancer substances [33,37]. This agrees with the results of a study conducted with the participation of 9271 patients, which showed an association between a high concentration of digitoxin in blood plasma and a low risk of developing malignant neoplasms of the blood and hematopoietic organs as well as a moderate decrease in the incidence of kidney cancer, urinary tract cancer and prostate cancer [88].

These retrospective observations are largely confirmed by in vitro studies, indicating the possibility of a direct inhibitory effect of CS on the proliferative and metabolic potential of various types of tumor cells [89,90]. For example, increased (compared with other tumors) expression of the α1-Na/K-ATPase subunit has been observed in non-small cell lung cancer, renal cell carcinoma, gliomas and melanomas and an increase in the α3-Na/K-ATPase subunit has been observed in colon cancer [91,92,93,94,95]. Several authors have noted a decrease in the content of the α1-Na/K-ATPase subunit observed in prostate cancer [96] while Kiss et al. suggested that the α1 subunit is a new target especially in the therapy of glioblastomas [97]. It is necessary to highlight that there is a significant increase in the intracellular concentration of Na+ and an increase in the content of Ca2+ in cells along with a moderate decrease in the intracellular concentration of K+ [92]. The effect of CS differ depending on the dose; thus, Li et al. demonstrated that in a human gastric cancer cell line (MGC803), bufalin at 20 nmol/L induced an M-phase cell cycle arrest whereas at 80 nmol/L, it induced apoptosis via an increased Bax/Bcl-2 ratio and activated caspase-3 [97]. These distinct effects correlated to the transient activation of the phosphatidylinositol 3-kinase (PI3K)/Akt signaling pathway [97]. Proscillaridin A was identified as a potential treatment compound with IC_50_ values ranging from 0.007 μM to 0.55 μM in various tumor types [98]. Importantly, the number of studies in which bufadienolides were used as an in vitro anti-cancer treatment has been heightened and bufadienolide inhibitors of the Na/K-ATPase that have been used in vitro and in vivo include MBG [71,72], bufalin [98,99], cinobufagin [100], resibufagenin [101], proscillaridin A [102], gamabufotalin [103] and 1α,2α-Epoxyscillirosidine [104]. When analyzing experiments and clinical data it is obvious that MBG and other Na/K-ATPase inhibitors hold promise to treat cancer and following anti-CS antibody treatment to PE patients we must expect a rise of Fli1 and be alert. The direct link between cancerogenesis, MBG and the activity of Fli1 is yet to be established.

## 7. Conclusions

It appears that the introduction of antibodies to MBG eliminated the inhibition of Na/K-ATPase in red blood cells obtained from the blood of patients with PE ex vivo [27,28]. In pregnant rats with experimental PE induced by the consumption of water with an excessive NaCl amount, the in vivo immunoneutralization of the MBG by poly and monoclonal antibodies exerted an anti-hypertensive effect associated with the restoration of vascular Na/K-ATPase activity [10]. We suggest that the interaction of MBG and Na/K-ATPase is the cornerstone in pathogenesis of PE. The development of PE is associated with an increase in MBG production, which through the Fli1-dependent mechanism stimulates the synthesis of collagen in the umbilical arteries and finally leads to the impairment of vasorelaxation and the development of vascular stiffness that may progress beyond PE [33,37]. Understanding of the role of Fli1 and MBG in the development of PE gives us a possibility to suggest CS as one of the therapeutic tools for the treatment of cancer. As the measurement of MBG has become available in plasma of pregnant subjects via sensitive analytical methods relying on liquid chromatography combined with mass spectrometry [105], the immunoneutralization of MBG may become an effective direction in the treatment of PE. Considering the growing evidence for the role of CS in the pathogenesis of cancer, the extracorporeal route of anti-CS antibodies may be a fine-drawn method of immunotherapy of PE.

## Abbreviations

CS	Cardiotonic steroids
EGRF	Epidermal growth factor receptor
ERK	Extracellular signal-regulated kinases
ETS	Erythroblast transformation specific family
Fli1	Friend leukemia integration 1 transcription factor
MBG	Marinobufagenin
SMAD	Proteins, main signal transducers for receptors of the transforming growth factor beta
Src	A family of proto-oncogenic tyrosine kinases
PKC	Protein kinase C
PLC	Phospholipase C
PE	Preeclampsia
TGF-β	Transforming growth factor beta 1
